# A stepped wedge cluster randomised trial of nurse-delivered Teach-Back in a consumer telehealth service

**DOI:** 10.1371/journal.pone.0206473

**Published:** 2018-10-31

**Authors:** Suzanne Morony, Kristie R. Weir, Katy J. L. Bell, Janice Biggs, Gregory Duncan, Don Nutbeam, Kirsten J. McCaffery

**Affiliations:** 1 Sydney School of Public Health, The University of Sydney, Sydney, Australia; 2 Health Literacy Lab, Sydney School of Public Health, The University of Sydney, Sydney, Australia; 3 Healthdirect Australia, Sydney, Australia; 4 Eastern Health Clinical School, Monash University, Box Hill, Australia; Nord University, NORWAY

## Abstract

**Objective:**

To evaluate the impact of Teach-Back on communication quality in a national telephone-based telehealth service, for callers varying in health literacy.

**Design:**

Cross-sectional stepped wedge cluster randomised trial with continuous recruitment, short (fixed) exposure and blinded outcome assessors. Nurses were stratified by hours worked and randomised into training groups using a computer generated sequence.

**Setting:**

An Australian national pregnancy and parenting telephone helpline.

**Intervention:**

Complex intervention involving a single 2-hour group Teach-Back training session, combined with ongoing nurse self-reflection on their communication following each call and each shift.

**Participants:**

Participants were 637 callers to the telephone helpline aged 18–75 (87% female), of whom 127 (13%) had inadequate health literacy (measured with the Single Item Literacy Screener); and 15 maternal and child health nurses with 15 years’ experience on average.

**Measures:**

Primary outcome was a modified subscale of the Health Literacy Questionnaire, ‘having sufficient information to manage health’. Secondary caller outcomes included caller confidence, perceived actionability of information and nurse effort to listen and understand. Nurse outcomes were perceptions of their communication effectiveness.

**Results:**

Over a 7 week period, 376 surveyed callers received usual care and 261 Teach-Back. Ratings on the primary outcome increased over time (OR 1.17, CI 1.01 to 1.32, p = 0.03) but no independent Teach-Back effect was observed. A consistent pattern suggests that, compared with usual care, Teach-Back helps callers with inadequate health literacy feel listened to (OR 2.3, CI 0.98 to 5.42, p = 0.06), confident to act (OR 2.44, CI 1.00 to 5.98, p = 0.06), and know what steps to take (OR 2.68, CI 1.00 to 7.17, p = 0.06). Nurse perceptions of both their own communication effectiveness (OR = 2.31; CI 1.38 to 3.86, p<0.0001), and caller understanding (OR = 2.56; CI 1.52 to 4.30, p<0.001) both increased with Teach-Back. No harms were reported.

**Conclusions:**

Teach-Back appears to benefit telephone health service users with inadequate health literacy, but the extent of this is unclear due to smaller numbers of lower literacy participants. Improving caller ratings over time are likely due to increasing nurse communication competence.

## Introduction

### Background

Telephone based telehealth services offer a convenient, cost-effective, and accessible way to provide health information and instructions[1] yet also carry the risk that callers may misunderstand the information they have been given[2]. (Even in face-to-face consultations) patients may misunderstand or forget what they have been told or fail to adhere to instructions [3–5]. This is particularly problematic for people with low health literacy, who are less likely to ask questions or indicate when they do not understand [6–12]. Telephone health providers can send supplementary written information electronically; however, for people who have difficulties reading (in English) or accessing information online this may be insufficient support. Electronically mediated health services including telehealth may have potential to worsen health disparities if people at risk of social health inequalities lack knowledge of telehealth services and how and when to use them[13].

A key communication technique to improve patient understanding and safety is Teach-Back [4, 5, 14–17]. Teach-Back is a process whereby the health information provider (e.g. nurse) takes on the burden of ensuring effective communication e.g. by saying “I want to make sure I have explained everything correctly”, and asks the patient to restate the information or advice they have been given, using their own words [18]. The clinician can then correct any misunderstandings or add reminders of important actions to take if required [18]. Previous randomised controlled trials have found Teach-Back effective in improving emergency department discharge [19] and obtaining informed patient consent [20, 21]. Teach-Back has been used in outpatient management [16], to educate, assess learning and improve recollection of health information [22], and improve accuracy of laboratory results communicated between professionals by telephone [23]. It was recently ranked the number one health literacy practice by experts [17] and may also help health services improve client satisfaction and meet expectations [24].

Despite recommendations to use Teach-Back for telephone consultations, [1] [2] to our knowledge there are no previous efforts to systematically evaluate Teach-Back in a telephone based telehealth service. Teach-Back has sometimes been difficult to implement in health services, largely due to perceptions that it can increase consultation time, healthcare provider discomfort and concerns with phrasing, and quantity and complexity of information to discuss. We have discussed these issues and detailed the intervention we designed to address them elsewhere [25].

We aimed to implement Teach-Back in a telephone service providing information and advice for pregnancy and parenting of young children, and evaluate the impact on caller ratings of information they received and experience of the call. Trial and training session duration were constrained, and it was desirable for all participating nurses to receive the intervention. We designed a complex intervention to address key barriers to using Teach-Back and maximise nurse self-directed learning (see[25]) which we implemented in a stepped wedge RCT design. We have reported nurse and caller experiences with Teach-Back elsewhere[26].

## Methods

### Setting

The Pregnancy Birth and Baby (PBB) helpline is a government-funded national telephone service offering free information and advice on pregnancy and parenting (up to 5 years), staffed by qualified maternal and child health nurses at a single call centre. Nurses use computers to record caller data and access and forward online information; but can leave their desks using wireless headsets.

### Study design

We conducted a cross-sectional stepped wedge cluster randomised trial with three-steps, continuous recruitment, and short (fixed) exposure [27]. Participants were callers to the helpline, recruited by helpline nurses (clusters). The intervention was nurse training in Teach-Back (detailed in [25]), and outcomes (impact of Teach-Back) were self-report measures of callers and nurses.

**Fig 1:**
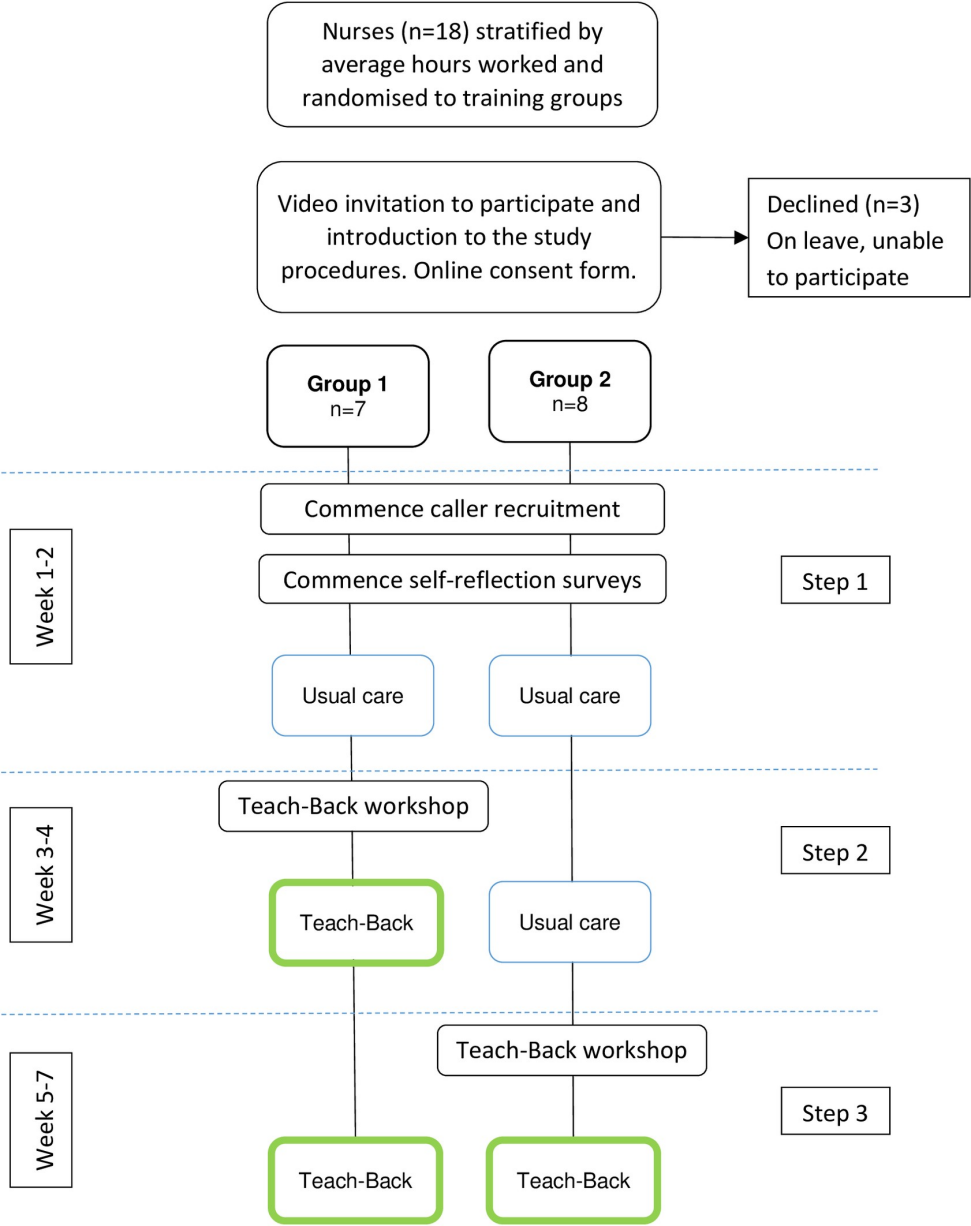
Flow chart of nurse (cluster) study activities.

Initially all nurses were in the unexposed condition; following Teach-Back training, nurses crossed over to the exposed condition (Fig 1). This design was chosen for pragmatic reasons: firstly so that all participating nurses could be trained in Teach-Back; and secondly to minimise the crossover period and possibility of contamination.

### Trial registration

Australian New Zealand Clinical Trials Registry, ACTRN12616000623493. Retrospectively registered.

### Patient involvement

The trial was developed as part of a wider health literacy initiative at Healthdirect Australia, aiming to improve patient care. Survey questions were piloted with a small (highly educated) convenience sample of parents and expecting parents. Study participants were invited to receive study feedback by email.

### Piloting and field testing

Several weeks prior to study commencement JB and DZ listened to a subset of calls and confirmed that Teach-Back was not currently being used, and that calls shorter than 4 minutes duration were unsuitable for Teach-Back. Following piloting (see above), the survey was field tested by a third party survey company (Population Research Laboratory (PRL)) with a sample (n = 20) of helpline callers.

### Participants

#### Callers

Consenting callers were eligible if they were at least 16 years old and fluent in English. Exclusions were calls shorter than 4 mins duration, sensitive topic (e.g. pregnancy or child loss, family violence), caller distress, nurse not participating, or caller transferred to other services. Telephone numbers were screened by PRL to exclude callers already recruited into the study.

#### Nurses

All nurses who were rostered to work on average more than 3 hours per week during the study period (n = 18) were invited to participate.

#### Recruitment

For callers we obtained informed oral consent at two points. This procedure was approved by the University of Sydney Human Research Ethics Committee (2016/083) and accepted by the Royal District Nursing Service Ethics Committee (approval number 150013). Oral consent to be contacted for follow-up was recorded using the same system regularly used by Healthdirect Australia to obtain consent for surveying client satisfaction. We amended the consent script to make it clear that the data collected during this study would be used for research purposes. Consent for follow-up is recorded on IT systems managed by Healthdirect Australia. Caller contact details for follow-up were only available to the researchers for those callers who had provided oral consent.

Approximately one week after their initial helpline call, consenting callers were contacted by trained female telephone interviewers and invited to participate in the research. Before the interview could commence callers were asked to consent to be interviewed. They were informed the survey was voluntary and their information would be kept confidential.

Written nurse consent forms were distributed and completed electronically.

### Intervention

Training in theory and skills for using Teach-Back was a 2-hour “communication skills” workshop (7–8 nurses per group), 2 or 4 weeks after study commencement. During the workshop trainers and nurses discussed how to use Teach-Back for different kinds of calls at the helpline, and acknowledged that not all calls would be suitable for Teach-Back. For example, some callers may simply want to confirm with a nurse that a course of action they have decided on (e.g. take a child to the doctor) is appropriate; Teach-Back would be unnecessary and inappropriate in such situations. For the duration of the study, nurses were encouraged to reflect after each call on how effectively they communicated and how well the caller understood. This is detailed in [25] and summarised in Fig 1.Emails encouraging participation and reiterating study procedures were sent to both nurse groups, in addition to individualised responses to any questions.

### Outcomes

#### Callers

Caller outcomes were assessed in a single telephone survey conducted by PRL approximately one week following initial contact. The primary outcome was “having sufficient information to manage health”, a subscale of the Health Literacy Questionnaire (HLQ) [28]. This scale is comprised of 4 likert-type items with 4 response categories. We used a modified version that replaced references to “health” with terminology suited to pregnancy and parenting issues (S1 Text). Secondary outcomes are caller satisfaction [29], confidence to manage the issue (adapted from [30] [31]), actionability of advice (developed from the Patient Education Materials Assessment Tool [32] actionability component), expectations [29], self-reported adherence to advice, and feeling listened to and understood (2 items from the CollaboRATE measure of shared decision-making[33]). Several measures were adapted for telephone by capitalising words to be emphasised and moving scale anchors for consistency across scales. A complete list of variables and scales is detailed in S1 Table. To examine the impact of Teach-Back for participants with low health literacy, we assessed callers’ health literacy level using the Single Item Literacy Screener (SILS) [34], which asks how frequently adults need help reading written health material. Health literacy was dichotomised as adequate (never, rarely, sometimes)/inadequate (often, always).

#### Nurses

Nurse measurements were two self-reported items on a five-point scale: “How effective do you think you were at communicating the information” and “How well do you think the caller understood your instructions or recommendations?”; with responses recorded in an online survey following each call (see [25]). In the Teach-Back condition, surveys also captured whether nurses reported using Teach-Back[25].

### Sample size

Calculations indicated a sample of 400 callers (200 receiving Teach-Back and 200 receiving usual care), would be sufficient to detect a difference of at least 0.5 standard deviation (SD)[35] in the mean total score for the HLQ subscale ‘having sufficient information to manage health” [28, 36] with 80% power at the two-sided 5% significance level, assuming 16 clusters (nurses), a minimum of 5 participants (callers) per cluster per step, unequal cluster size, estimated 80% response rate and intention to treat analysis. To mitigate against possible nurse dropout or non-participation, we oversampled, aiming for a total sample of n = 600.

The trial was designed to run over 6 weeks. Numbers recruited in the usual care condition were higher than anticipated so the trial was extended by an additional week to balance the groups.

### Randomisation and blinding

#### Callers

Callers to the PBB Helpline (participants) were invited by the nurse who took their call to participate in a survey for quality, service improvement, and research purposes. The Teach-Back trial was not explicitly disclosed to callers. Nurses used the same recruitment script in both conditions. Callers were allocated to condition according to whether the recruiting nurse had attended Teach-Back training (Fig 1). Caller interviewers (PRL) were blinded to the study design, allocation, and intervention.

#### Nurses

Eligible nurses were stratified (into 4 strata) based on average number of hours rostered per week (to balance the number of calls to each condition), and randomly allocated to one of two training groups by SM using the RAND function in Excel. Nurses were invited to participate via a video introduction to the study and online consent form. They were advised they had been randomised into training groups for a “communication skills workshop” on a specific date. To minimise contamination nurses were asked not to discuss the content of training outside of their group and to avoid sharing training materials until both groups had completed training.

### Statistical analysis

Analysis was by intention to treat. Due to highly skewed distributions of both caller and nurse data on most non-categorical outcomes we dichotomised all outcome variables into “highest scale category” and “other”.

We operationalised effects of time as week (1–7) of initial call to the helpline (measured as date of call for caller outcomes or date of self-reflection for nurse outcomes) and examined the data for an underlying time trend [37]. We used non-linear mixed effects models (logistic regression; NLMIXED procedure in SAS 9.4) to estimate the effects of Teach-Back on multiple outcomes that reflected (i) caller perceptions of advice (single measurements per caller) (ii) nurse perceptions of communication (repeated measurements per nurse over the course of the trial).

Models for the caller data were adjusted for time and health literacy, and included random intercepts for nurses to account for clustering of callers [37, 38]. We used Likelihood Ratio Tests and Z-scores to test for statistical significance of main effects and pre-specified subgroup analyses within health literacy subgroups respectively. Confidence intervals were calculated using the models’ beta coefficients and standard errors, assuming a normal distribution on the log-scale, with back-transformation to the natural scale (i.e. LCL = exp^(beta coeff –1.96×SE)^ and UCL = exp^(beta coeff+1.96×SE)^). The intra cluster coefficient (ICC) was calculated as (variance of random intercepts / [variance of random intercepts + π^2^/3]). We analysed whether Teach-Back effects on caller perceptions differed between nurses (Teach-Back random effects) and if they changed over time (Teach-Back ^×^ time interactions), allowing for covariance parameters with the random intercepts (baseline differences between nurses), but set this to zero if required for model convergence.

For nurse self-reflections, we included random intercepts for nurses to account for clustering of repeated measurements within nurses and tested for both fixed and random effects of time and Teach-Back, and for Teach-Back ^×^ time interactions.

## Results

### Participant flow

**Fig 2:**
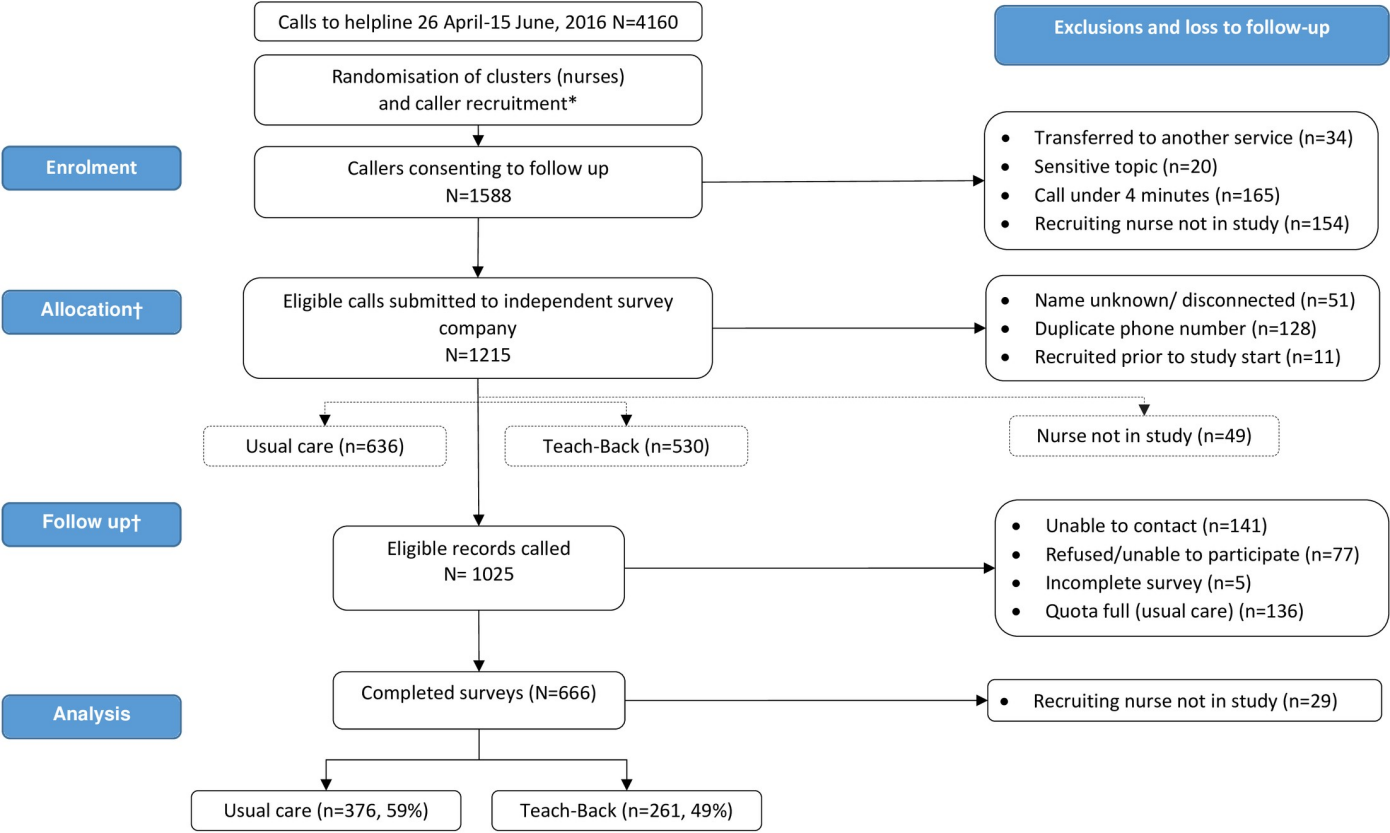
Flow chart of calls.

Eighteen eligible nurses were identified and randomised to training groups; three did not participate due to rostering or leave. Nurses recruited 1588 callers between 26 April and 15 June, 2016, of which 1215 were submitted to the survey company (Fig 2). After removing duplicates and wrong numbers there were 1025 eligible calls, for which 666 caller surveys were completed. Of these, 29 were excluded because the recruiting nurse did not participate in the study. The overall response rate was calculated as 65.5%, taking into account partially completed surveys (n = 5, not included in analyses). This is acceptable [39]. In the final sample (n = 637), 376 were in the usual care (59% completion rate) and 261 in the Teach-Back condition (49% completion rate). These completion rates are underestimates and do not account for the 190 calls that were duplicates or wrong numbers; we do not know allocation for those calls.

### Recruitment

Recruitment of callers to the study was highly variable across nurses (detailed in Table 1) and reflected differences in the case load for each nurse. The number of eligible callers decreased each week as repeat callers were excluded. Two nurses recruited fewer than 15 surveyed callers, and more than half the participating nurses (n = 9) recruited fewer than 5 surveyed callers at each “step” in the study. The coefficient of variation in cluster size (CV)[40, 41] (standard deviation of cluster size divided by mean cluster size), was 0.89. Time between initial helpline call and follow-up survey ranged from 2–38 days (mean 9; median 8); IQR = 4.

**Table 1:** Number of cases (callers) recruited at each stage of the trial. Shading indicates the clusters (nurses) were exposed to the intervention.

		Number of callers surveyed per step* (Number of eligible callers recruited per step)			Total callers
Training group	Cluster (nurse)	Step 1 Weeks 1–2	Step 2 Weeks 3–4	Step 3 Weeks 5–7	
Group 1	1	6	7	4	17
		(13)	(12)	(10)	(35)
	2	29	15	20	64
		(52)	(38)	(47)	(137)
	3	11	2	3	16
		(14)	(6)	(5)	(25)
	4	7	7	11	25
		(12)	(15)	(25)	(52)
	5	44	37	2	83
		(78)	(38)	(10)	(159)
	6	5	0	2	7
		(6)	(1)	(4)	(11)
	7	9	3	12	24
		(13)	(13)	(20)	(46)
Group 2	8	7	1	8	16
		(16)	(4)	(20)	(40)
	9	9	3	9	21
		(14)	(4)	(16)	(34)
	10	49	44	47	140
		(90)	(65)	(80)	(235)
	11	12	15	16	43
		(15)	(33)	(34)	(82)
	12	26	32	30	88
		(55)	(51)	(51)	(157)
	13	16	25	14	55
		(22)	(38)	(29)	(89)
	14	3	2	1	6
		(4)	(4)	(2)	(10)
	15	14	7	11	32
		(22)	(11)	(21)	(54)
	Cluster unexposed to intervention				376 (636)
	Cluster exposed to intervention				261 (530)
**Total**					
Group 1 (clusters 1–7)		111 (188)	71 (156)	54 (121)	236 (465)
Group 2 (clusters 8–15)		136 (238)	129 (210)	136 (253)	401 (701)
*Nurses not participating (n = 6)*		*19* *(32)*	*10* *(17)*	*0*	*29* *(49)*
Grand total		266 (458)	210 (383)	190 (374)	666 (1215)†

* Power calculations assume a minimum of 5 calls per cluster per step

^†^ This number includes callers who gave a wrong number, and duplicate numbers. The final number of eligible calls was 1025, detailed in Fig 2.

### Demographics and descriptive statistics

Caller characteristics are detailed in Table 2. Callers were predominantly female (87%), university educated (64%) and had adequate health literacy (80%). Almost half (48%) identified as the mother of the subject of the call, 12% as a father or male partner, and 36% as the subject. Call topics could be broadly classified as antenatal (30%) and postnatal (70%) issues.

**Table 2:** Caller demographic characteristics for whole sample and inadequate (low) health literacy.

	Total N = 637		Usual care N = 376		Teach-Back N = 261	
	Mean	SD	Mean	SD	Mean	SD
Age	31.33	6.48	31.48	6.56	31.11	6.36
	N	%	N	%	N	%
Health literacy*						
Inadequate	127	19.9	67	17.8	60	23.0
Caller gender						
Female	552	86.7	338	89.9	214	82.0
*Low health literacy*, *Female*	*104*	*81*.*9*	*57*	*85*.*1*	*47*	*78*.*3*
Language spoken at home						
Language other than English	208	32.7	116	30.9	92	35.2
*Low health literacy*, *Language other than English*	*68*	*53*.*5*	*37*	*55*.*2*	*31*	*51*.*7*
Education						
High school (incomplete)	7	1.1	3	0.8	4	1.5
High school (complete)	135	21.2	85	22.6	50	19.2
Vocational education	87	13.7	46	12.2	41	15.7
University	407	63.9	241	64.1	166	63.6
*Low health literacy*, *University*	*77*	*60*.*6*	*42*	*62*.*7*	*35*	*58*.*3*

* Single Item Literacy Screener (SILS):”In general, how often do you need to have someone help you when you read instructions, pamphlets or other written material from your doctor or pharmacy? Would you say: never, rarely, *sometimes*, *often or always*?” (Italicised response options indicate inadequate health literacy [34])

Around 33% of callers spoke a language other than English at home, which is higher than 2016 population estimates (22%)[42]. Of these, 81% had attended university, possibly reflecting greater service uptake among skilled migrants. Given the high associations between education and language spoken at home, we did not conduct planned subgroup analyses for language spoken at home.

The 15 participating nurses (clusters) were all female, aged 37–61 years (mean 54.9) and had been registered as nurses for 13–41 years (mean 28.8 years) and working in maternal and child health for between 7 months and 33 years (mean 15 years). Two had worked on another helpline previously. Workload per week per cluster ranged from a single shift (3–4 hours) to 4 days (32 hours). Demographic information is unavailable for 3 nurses who worked a small number of shifts.

### Caller outcomes

Descriptive statistics for the dichotomised responses on each caller outcome in Teach-Back and usual care groups, both overall and by health literacy subgroup, are presented in Table 3. These indicate that ratings of the helpline were high overall; and that within conditions (i.e. usual care or Teach-Back) ratings tend to be higher among participants with adequate health literacy. For the primary outcome “having sufficient information to manage health” (dichotomous scale) the ICC was calculated at 0.019. The results of the mixed effects models analysis (Table 3) are summarised below: temporal trends first, followed by Teach-Back.

**Table 3:** Descriptive and model results for caller outcomes. All analyses adjusted for time and health literacy.

		Descriptive results				Model results		
		n	Possible scores†	% (n) in highest category		Odds ratio*	(95% CI)	P value†
**Primary outcome **				**Usual care**	**Teach-Back**			
*Having sufficient information to know what to do*								
Teach-Back, **overall**		628	1–4	40.2 (150)	45.5 (116)	0.77	0.44 to 1.37	0.37
Teach-Back, by **health literacy**								*0*.*44†*
	Inadequate	125		31.3 (21)	41.4 (24)	1	0.42 to 2.39	0.40
** **	Adequate	503		42.2 (129)	46.7 (92)	0.72	0.39 to 1.32	0.23
**Satisfaction**	** **							
*How USEFUL was the information or advice you received during your call*?								
Teach-Back, **overall**		636	1–5	70.7 (266)	72.3 (188)	0.77	0.41 to 1.45	0.4
Teach-Back, by **health literacy**								*0*.*75†*
	Inadequate	127		73.1 (49)	76.7 (46)	0.83	0.32 to 2.18	0.37
** **	Adequate	509		70.2 (217)	71.0 (142)	0.76	0.40 to 1.46	0.28
**Confidence to manage the situation**								
*How confident were you in knowing how to manage the issue BEFORE you called the helpline*?								
Teach-Back, **overall**		635	1–5	3.5 (13)	3.8 (10)	1.07	0.28 to 4.07	1.00
Teach-Back, by **health literacy**								*0*.*44†*
	Inadequate	127		6.0 (4)	3.3 (2)	0.55	0.08 to 3.99	0.33
** **	Adequate	508		2.9 (9)	4.0 (8)	1.39	0.33 to 5.86	0.36
*And AFTER speaking to the nurse*, *how confident were you in knowing how to manage the issue*?								
Teach-Back, **overall**		634	1–5	58.7 (219)	69.3 (181)	1.38	0.78 to 2.46	0.25
Teach-Back, by **health literacy**								***0*.*10****†*
	Inadequate	127		53.7 (36)	76.7 (46)	**2.44**	**1.00 to 5.98**	**0.06**
** **	Adequate	507		59.8 (183)	67.2 (135)	1.19	0.65 to 2.17	0.34
*How confident would you be in recommending the advice you were given to a friend who experiences similar problems*?								
Teach-Back, **overall**		629	1–5	64 (238)	67.7 (174)	1.08	0.60 to 1.96	0.75
Teach-Back, by **health literacy**								*0*.*53†*
	Inadequate	127		52.2 (35)	63.3 (38)	1.34	0.56 to 3.17	0.32
** **	Adequate	502		66.6 (203)	69.0 (136)	1.01	0.54 to 1.89	0.40
**Caller expectations**								
*Was the information you were given what you EXPECTED to hear*?								
Teach-Back, **overall**		621	1–5	33.5 (122)	29.6 (76)	1.39	0.80 to 2.42	0.24
Teach-Back, by **health literacy**								*0*.*65†*
	Inadequate	122		28.6 (18)	23.7 (14)	1.22	0.50 to 3.01	0.36
** **	Adequate	499		34.6 (104)	31.3 (62)	1.44	0.81 to 2.58	0.19
**Acting on referrals **								
*Did you contact the service/s*?								
Teach-Back, **overall**		297	Y/N/NY†	62.9 (112)	65.5 (78)	0.87	0.39 to 1.97	0.75
Teach-Back, by **health literacy**								*0*.*32†*
	Inadequate	65		72.5 (29)	64 (16)	0.55	0.16 to 1.87	0.25
** **	Adequate	232		60.1 (83)	66.0 (62)	1.01	0.43 to 2.41	0.40
**Actionability of advice **								
*The nurse told me all the steps I needed to take*.								
Teach-Back, **overall**		458	1–5	61.1 (160)	65.3 (128)	1.47	0.76 to 2.82	0.24
Teach-Back, by **health literacy**								***0*.*10****†*
	Inadequate	104		56.6 (30)	74.5 (38)	**2.68**	**1.00 to 7.17**	**0.06**
	Adequate	354		62.2 (130)	62.1 (90)	1.22	0.61 to 2.43	0.34
*The nurse checked that I fully understood the information*								
Teach-Back, **overall**		459	1–5	72.5 (190)	77.2 (152)	0.82	0.41 to 1.62	0.58
Teach-Back, by **health literacy**								*0*.*11†*
	Inadequate	104		58.5 (31)	76.5 (39)	1.45	0.54 to 3.86	0.30
** **	Adequate	355		76.1 (159)	77.4 (113)	0.65	0.31 to 1.35	0.20
*After talking to the nurse I felt sure that I knew what to do*								
Teach-Back, **overall**		461	1–5	68.8 (181)	75.8 (150)	1.08	0.52 to 2.25	0.75
Teach-Back, by **health literacy**								*0*.*21†*
	Inadequate	104		54.7 (29)	74.5 (38)	1.74	0.62 to 4.90	0.23
** **	Adequate	357		72.4 (152)	76.2 (112)	0.91	0.42 to 1.98	0.39
**Shared decision-making**								
*How much effort was made to listen to the things that mattered the most to you regarding the problem that you called about*?								
Teach-Back, **overall **		637	1–10*	66.2 (249)	72.4 (189)	1.34	0.77 to 2.47	0.32
Teach-Back, by **health literacy**								***0*.*09****†*
	Inadequate	127		49.3 (33)	70 (42)	**2.30**	**0.98 to 5.42**	**0.06**
** **	Adequate	510		69.9 (216)	73.1 (147)	1.12	0.60 to 2.06	0.37
*How much effort was made to help you understand the problem you called about*?								
Teach-Back, **overall**		633	1–10*	63.5 (238)	67.8 (175)	1.04	0.58 to 1.87	1.00
Teach-Back, by **health literacy**								***0*.*048****†*
	Inadequate	126		44.8 (30)	66.1 (39)	1.97	0.83 to 4.66	0.12
** **	Adequate	507		67.5 (208)	68.3 (136)	0.86	0.47 to 1.58	0.35
**Repeat callers**	** **							
*Have you or a family member called the Pregnancy*, *Birth and Baby helpline again since the initial call*?								
Teach-Back, **overall **		636		19.7 (74)	25.3 (66)	1.46	0.79 to 2.68	0.22
Teach-Back, by **health literacy**								*0*.*24†*
	Inadequate	127		28.4 (19)	43.3 (26)	**2.09**	**0.89 to 4.94**	**0.10**
** **	Adequate	509		17.9 (55)	19.9 (40)	1.25	0.64 to 2.42	0.32

*All models adjusted for week of call and health literacy

†p value for effect of Teach-Back for health literacy subgroups is for Teach-Back × health literacy interaction.

P values ≤ 0.10 bolded.

Notes: There was no evidence that the effect of Teach-Back differed between nurses for any of the outcomes. (Random effects of Teach-Back fitted assuming there was no correlation with random intercepts) There was no evidence that the Teach-Back effect changed over time for any of the outcomes (Teach-Back by time interactions fitted).

#### Temporal trend

Caller perceptions of information sufficiency (primary outcome) increased each week as the trial progressed (OR 1.17, CI 1.01 to 1.32, p = 0.03), as did Actionability of advice “the nurse checked that I fully understood the information” (OR 1.16, CI 0.99 to 1.37, p = 0.05). Caller expectations “was the information you were given what you expected to hear” also changed over time, (OR 0.86, CI 0.75 to 0.98, p = 0.03); qualitative responses indicated later callers found it *better* than expected. We found no other statistically significant effects of time.

#### Primary outcome–Having sufficient information to know what to do

After accounting for the time trend we found no incremental main effect of Teach-Back (OR 0.77, CI 0.44 to 1.37, p = 0.37) on the primary outcome, and no differences between the health literacy subgroups (p = 0.44; Table 3).

#### Secondary outcomes

After accounting for time we found no main effects of Teach-Back. For several outcomes there was some evidence that callers with inadequate health literacy in the Teach-Back condition gave higher ratings compared to those receiving usual care; and modest evidence of a more positive impact of Teach-Back for callers with inadequate health literacy. This pattern of differences held for outcomes relating to: Confidence, “after speaking to the nurse, how confident were you in knowing how to manage the issue?” (OR = 2.44, 95% CI 1.00 to 5.98, p = 0.06 [Teach-Back vs usual care for participants with inadequate health literacy]; vs OR = 1.19, p = 0.10 [Teach-Back participants with adequate heath literacy]); Actionability, “the nurse told me all the steps I should take” (OR 2.68, 95% CI 1.00 to 7.17, p = 0.06; vs OR = 1.22, p = 0.10); and Shared decision-making, “how much effort was made to listen to the things that mattered the most to you regarding the problem that you called about” (OR = 2.30, 95% CI 0.98 to 5.42, p = 0.06; vs OR = 1.12, p = 0.09), and “how much effort was made to help you understand” (OR 1.97, 95% CI 0.83 to 4.66, p = 0.12; vs 0.86 for inadequate vs adequate health literacy, p = 0.048 for subgroup difference in effect). There was modest evidence suggesting that participants with inadequate health literacy in the Teach-Back condition were more likely to have called the helpline again (since the initial call) than those receiving usual care (OR 2.09, 95% CI 0.89 to 4.94, p = 0.10). We found no evidence of difference in outcomes between Teach-Back and usual care conditions for callers with adequate health literacy.

#### Ancillary analyses

There was no evidence that the effect of Teach-Back differed between nurses nor that it changed over time for any of the outcomes (p>0.2 for random effects of Teach-Back, assuming no correlation with random intercepts; p>0.2 for all Teach-Back by time interactions). There was some evidence that the outcome “The nurse told me all the steps I needed to take” differed between nurses at different time points in the study (p = 0.03 for testing random effects of time, assuming no correlation with random intercepts) but this did not materially change the estimated effect of Teach-Back. There was no evidence that time effects differed between nurses for any of the other outcomes (all p > 0.2).

### Nurse outcomes

The online self-reflection surveys (Table 4) gathered a total of 1861 responses over a period of 7 weeks; 1005 in usual care and 856 in Teach-Back. The two self-reflection questions were highly correlated (Pearson’s r = .88, p < .001). Completion of the survey was inconsistent, and number of call reflections per nurse ranged from 14 to 608. This is partly due to differences in the number of shifts each nurse worked during the trial period and differences in interpretation of the instructions to self-reflect. It may also reflect engagement with the study and perceived importance of self-reflection activities. The total number of responses per cluster per condition was generally balanced, although 3 clusters (<45 responses per cluster) exhibited a highly skewed response distribution (i.e. >80% of their self-reflections were in one condition).

**Table 4:** Effects of Teach-Back intervention on nurses (n = 15). All analyses by intention to treat.

		Estimated odds ratio*	(95% CI)	P value†
*How effective do you think you were at communicating the information? *(1–5)*				
Teach-back		2.31	1.38 to 3.86	**<0.001**
	At week 3–4	1.40	0.78 to 2.52	**<0.001**
	At week 5–7	4.69	2.51 to 8.76	
*How well do you think the caller understood your instructions or recommendations? *(1–5)*				
Teach-back		2.56	1.52 to 4.30	**<0.001**
	At week 3–4	1.57	0.88 to 2.80	**<0.001**
	At week 5–7	6.56	3.39 to 12.69	

†p value for effect of Teach-Back for different weeks is for Teach-Back × time interaction.

*Questions were asked on a 5-point likert scale (extremely–not at all). After inspection of the distributions responses were dichotomised into “extremely” and “other”

Notes: Models include random intercepts and random time effects, and allow for correlation between the two random effects. There was also strong evidence that the effect of Teach-Back differed between nurses for both the outcomes (p<0.001 for Teachback random effects on both outcomes; models allowed correlation with random intercepts). These random effects are not reported in the Table above, but were included in the models.

There was very strong evidence that Teach-Back improved nurse perceptions both that their communication was effective (OR = 2.31; CI 1.38 to 3.86, p<0.0001), and that the caller understood them (OR = 2.56; CI 1.52 to 4.30, p<0.001); the magnitude of these effects differed between nurses (p<0.001 for Teach-Back random effects for both outcomes, fitted allowing for correlation with random intercepts). There was also very strong evidence that the effects of Teach-Back increased with time (OR = 1.40 vs 4.69 and OR = 1.57 vs 6.56 for effects at weeks 3–4 vs 5–7 for effectiveness of communication and caller understanding respectively; p<0.001 for Teach-Back×Time interactions for both outcomes).

#### Contamination/carry over effects

Some participants called the helpline more than once during the trial period, and could have received both Teach-Back and usual care. Although no caller was surveyed more than once, some repeat callers may have answered the survey questions with respect to a different call, meaning assignment to condition (Teach-Back or usual care) could be incorrect. We searched the database of eligible calls (n = 1215) for repeat phone numbers: almost 20% of callers (n = 220) called more than once during the study period, and usually spoke with a different nurse. Of 637 surveyed callers, 58 (9%) called more than once during the study period: 39 (6.1%) called twice, and 19 (3%) called 3 times or more (maximum 9). Time between (adjacent) calls ranged from 0 to 49 days (median 6.5).

In focus groups and interviews following the trial (reported in [25] [26]) nurses reported minimal contamination during the crossover period, also noting they rarely heard each other talking with callers. This suggests Teach-Back is unlikely to have been used in the usual care condition; however, evidence from nurse self-reports indicates Teach-Back was not always used in the Teach-Back condition. Its impact may therefore be underestimated in the data.

#### Harms

No harms to nurses or callers were identified in this study.

## Discussion

To our knowledge this trial is the first attempt to systematically evaluate Teach-Back in a telephone helpline, and it adds to the growing evidence base on the use of Teach-Back to improve healthcare communication, particularly for consumers with lower health literacy [19, 43–45]. We used a structured intervention (nurse self-reflection and Teach-Back skills training—detailed in [25]) and randomised stepped wedge study design. The response rate was adequate (65.5%), although some assumptions of the power calculations were not met.

Descriptive statistics suggest a positive effect of Teach-Back on a number of caller outcomes, including the primary outcome; however, a strong temporal trend in the data accounted for much of this variance. Although there was no independent effect of Teach-Back on the primary outcome (having sufficient information to manage health) our analysis of secondary outcomes identified that Teach-Back can increase perceptions relevant to knowing what to do to manage health for callers with inadequate health literacy. This includes perceptions of good communication practice (i.e. feeling listened to and helped to understand), confidence to know what to do, and knowing what steps to take. There is potential to improve child health outcomes if Teach-Back can improve parent comprehension of information [46]. A small number of callers who participated in qualitative interviews expressed mostly positive experiences with Teach-Back (see [26] for detailed report). Nurses’ perceptions of their own communication effectiveness also increased with the use of Teach-Back.

Prior work has often reported that health providers can be reluctant to use Teach-Back due to perceptions it can increase consultation time [24, 43, 47–49]. We did not report impact on call duration, because during the study period nurses were encouraged to focus on practising Teach-Back skills rather than meeting call duration targets, and skill learning may have temporarily inflated average call duration. Call duration is an important metric for call centres and it will be important to examine this in future work. Some nurses self-reported that Teach-Back helped to close an interaction with a caller[26]; previous work also indicated Teach-Back can help manage consultation time [50].

We estimate (from self-reflection data) that around 30% of calls in the Teach-Back condition did not involve Teach-Back, and in In focus groups and interviews (reported in [26] [25]) nurses commented that this was sometimes because they forgot, did not feel Teach-Back was appropriate, or did not know how to use Teach-Back for that kind of call. This is a reflection on the short time frame for training and limited opportunities for nurses to learn from each other during the study period. It also implies that the impact of Teach-Back is likely to be underestimated in these data.

Given that caller measurements were taken as nurses were learning new skills, the significant interaction between Teach-Back and time is likely due to a “rising tide effect” [51] rather than calendar time. Caller perceptions of the care they received was likely improved by nurses’ purposeful reflection on how well they explained things, how well they thought the caller understood, and on the communication techniques that worked well for them. Nurse practice in self-reflecting and using Teach-Back may account for improvements over time in both caller ratings (that information was sufficient, that the nurse confirmed their understanding, and information was better-than-expected) and nurse self-ratings.

### Strengths and weaknesses of the study

This study was embedded into a quality and safety improvement activity, using a randomised design and concealed allocation with low potential for contamination. The stepped wedge design used is a strength in terms of sample size efficiency and practicality of implementation; however the trial did not meet all assumptions of the power calculations. Unlike previous work[19] we had no objective measure of client comprehension or recall of information (which varied on every call so it was not possible to assess it objectively and uniformly across the sample), and a time delay between the consultation and data collection, which may have contributed to a lack of statistical effect.

A weakness is the lack of a true measure of “usual care” at baseline and the short timeframe for data collection. All measurements were taken after nurses had commenced self-reflection, which appeared to have an independent effect on caller outcomes. Combined with practice or learning effects, it may have diluted the Teach-Back effect in the data. This effect would be further diluted by the fact that not every caller surveyed in the Teach-Back condition received Teach-Back. The lower survey completion rate in Teach-Back also reflects the shorter timeframe for follow-up. Future work that can address these shortcomings may produce a more true measure of the impact of Teach-Back.

Inadequate health literacy was operationalised with a measure of needing help reading health information that is moderately effective at identifying patients with limited literacy [52], and captures those callers who are unable to benefit from supplementary written information sent by email. The proportion of people identified as having inadequate health literacy (20%) is within range of previous Australian health literacy surveys using telephone recruitment (7–26% [53]; 24%, [54]); however the relatively small number of surveyed callers with inadequate health literacy made it more difficult to detect differences for this important subgroup.

### Implications

Although the results of this study were not definitive, the balance of evidence reported here (and [25, 26]) supports the use of Teach-Back by health service providers in call centres of this nature. It may also have useful applications in other areas of telehealth. Consumers with lower health literacy in particular appear to benefit from Teach-Back and we have no evidence for any negative impact on caller outcomes, including satisfaction. The technique used to facilitate nurse development of Teach-Back skills (i.e. active self-reflection on their communication) appears to improve both provider and caller perceptions of the quality of communication. This might play a useful role in development of other telephone health communication skills and is a worthwhile topic for future studies. As discussed above, impact on call duration (and by extension impact on cost of service provision) could not be reliably ascertained and should be examined in future research.

It is an ongoing challenge for helpline providers to reach and support consumers with lower health literacy, who typically have poorer access to written health information [55, 56] and rely more on verbal reinforcement. They may also have poorer knowledge of available telehealth services and lack of confidence using the telephone [57]. In Australia, many regional and remote communities have limited access to mainstream health services due to distance, and stand to benefit from telehealth. Our study suggests that systematically implementing Teach-Back in a telephone based telehealth service may help to support people with low health literacy, and help reduce inequalities in the provision of telephone health information.

### Unanswered questions and future research

Studies of this type (real-time, real world conditions) present methodological challenges in managing exposure and consistency in relation to the intervention, and in measuring change in primary outcomes. In a trial of longer duration, introducing a “transition” period at each step during which the cluster would be considered neither exposed not unexposed [51], and an objective measure of implementation fidelity [58] may help to isolate the specific effects of Teach-Back. This could also inform about the impact of Teach-Back on call duration.

We do not have objective data on whether callers understood correctly or followed through on recommended actions, and it is possible some callers may have acted incorrectly on information they misunderstood, or only acted on part of the information. Some previous work suggests callers may sometimes think they have acted in accordance with advice when in fact they did not [59, 60]. Future work to improve access to telehealth services for people with low health literacy could include teaching telephone communication skills to adult learners at risk of low health literacy (see e.g. [57]) to potentially improve uptake.

## Conclusion

Teach-Back appears to have promise for improving communication of health-related information in a consumer helpline. More work is needed to explore whether the systematic use of enhanced communication techniques such as Teach-Back is effective in different telehealth contexts.

## Supporting information

S1 Text Adaptations to Heath Literacy Questionnaire (primary outcome). (DOCX)

S1 Table Complete list of registered secondary outcome measures and scale reliability and inter-item correlations. (DOCX)
