# Supplementary material for: A stepped wedge cluster randomised trial of nurse-delivered Teach-Back in a consumer telehealth service
Source: PLoS One. 2018 Oct 31;13(10):e0206473. doi: 10.1371/journal.pone.0206473 (PMC6209310; doi:10.1371/journal.pone.0206473)
Supplement: S1 Text — (DOCX) [file pone.0206473.s001.docx]

S1 Text: Adaptations to Heath Literacy Questionnaire (Primary outcome)

Adaptations to Heath Literacy Questionnaire (capitals for emphasis in telephone script)

Scale

1. Strongly disagree
2. Disagree
3. Agree
4. Strongly agree

4.1 I feel that I now have GOOD information about this issue.

4.2: I have ENOUGH information to help me deal with this problem.

4.3: I am sure I have all the information I need to MANAGE this issue EFFECTIVELY.

4.4: I have all the information I need to CARE for my pregnancy or child on this issue.
