# Supplementary material for: A stepped wedge cluster randomised trial of nurse-delivered Teach-Back in a consumer telehealth service
Source: PLoS One. 2018 Oct 31;13(10):e0206473. doi: 10.1371/journal.pone.0206473 (PMC6209310; doi:10.1371/journal.pone.0206473)
Supplement: S1 Table — (DOCX) [file pone.0206473.s002.docx]

S1 Table: Complete list of registered outcome measures and scale reliability and inter-item correlations.

| Scale/item (scale points) | Sourced from | Alpha Reliability* | Inter-item correlation* |
| --- | --- | --- | --- |
| 1. *Having sufficient information to manage health* |  |  |  |
| Scale (4 items) adapted for this study (see S1) | Health Literacy Questionnaire | **.938** | **.711-.882** |
| 1. *Caller satisfaction* (1-5). | **S1** |  |  |
| Overall, how satisfied were you with the service provided by the Pregnancy, Birth and Baby helpline? | Question used previously in evaluation of this [56] and other telehealth service[29]. |  |  |
| 1. *Confidence (1-5)*. | **S2** |  |  |
| How confident were you in knowing how to manage the issue BEFORE you called the helpline? |  | n/a | n/a |
| And AFTER speaking to the nurse, how confident were you in knowing how to manage the issue? | Adapted from [30] (see also [31]) |  | **.649** |
| How confident would you be in recommending the advice you were given to a friend who experiences similar problems? | Modified item from the credibility/expectancy questionnaire [57] set 1 item 3 |  |  |
| 1. *Links to other services.* | *Fewer than half the respondents reported being referred to other services. These data are not reported here.* |  |  |
| Were you referred to any [of the following] services? | Questions were created for a previous customer satisfaction survey for the PBB Helpline and adapted for this study |  |  |
| 1. *Expectations* | **S4** |  |  |
| Was the information you were given what you EXPECTED to hear? | Adapted from [29] |  |  |
| 1. *Experiences during the call* |  |  |  |
| Did the information provided by the helpline answer your query? | Adapted from a previous customer satisfaction survey for the PBB Helpline |  | **.745** |
| How USEFUL was the information or advice you received during your call? | Adapted from [29] |  |  |
| 1. *Actionability* of advice (1-5). | **S6** Items were based on the PEMAT actionability scale [58], which measures how well patient information materials identify and explain actions to be taken. |  |  |
| The nurse told me all the steps I needed to take |  | **.801** | **.539-.666** |
| The nurse checked that I that I fully understood the information |  |  |  |
| After talking to the nurse I felt sure that I knew what to do. |  |  |  |
| 1. *Feeling listened to (shared decision-making)* (1-10). | **S7** Two items adapted for telephone from the CollaboRATE measure (normally 0-9) [33] |  |  |
| On a scale of 1-10 where 1 means no effort was made and 10 means every effort was made, how much effort was made to listen to the things that mattered the most to you regarding the problem that you called about? |  |  | **.790** |
| And using the same scale, how much effort was made to help you understand the problem you called about? |  |  |  |
| 1. Repeat callers. | Items developed for this study |  |  |
| Have you or a family member called the Pregnancy, Birth and Baby helpline again since the initial call?  Was this call about the same or a different issue? |  |  |  |

| 1. Nurse items | Real-time self-assessments of performance using methods developed for this study. Adapted from [59] | Item correlation |
| --- | --- | --- |
| How effective do you think you were at communicating the information? |  | .88 |
| How well do you think the caller understood your instructions or recommendations? |  |  |

*correlations and reliability coefficients generated for scale responses. These responses were dichotomised (highest category/other) for the outcomes analysis.

|  | HLQ1 | HLQ2 | HLQ3 | HLQ4 | SATS1 | CON1 | CON2 | CON3 | EXT1 | EXP1 | EXP2 | ACT1 | ACT2 | ACT3 | SDM1 | SDM2 |
| --- | --- | --- | --- | --- | --- | --- | --- | --- | --- | --- | --- | --- | --- | --- | --- | --- |
| HLQ1: I feel that I now have GOOD information about this issue. | 1.00 |  |  |  |  |  |  |  |  |  |  |  |  |  |  |  |
| HLQ2: I have ENOUGH information to help me deal with this problem. | **.834^**^** | 1.00 |  |  |  |  |  |  |  |  |  |  |  |  |  |  |
| HLQ3: I am sure I have all the information I need to MANAGE this issue EFFECTIVELY. | **.736^**^** | **.810^**^** | 1.00 |  |  |  |  |  |  |  |  |  |  |  |  |  |
| HLQ4: I have all the information I need to CARE for my pregnancy or child on this issue. | **.711^**^** | **.756^**^** | **.885^**^** | 1.00 |  |  |  |  |  |  |  |  |  |  |  |  |
| SATS1: Overall how SATISFIED were you with the service provided by the Pregnancy, Birth and Baby helpline? | **.511^**^** | **.520^**^** | **.508^**^** | **.473^**^** | 1.00 |  |  |  |  |  |  |  |  |  |  |  |
| CON1: How confident were you in knowing how to manage the issue BEFORE you called the helpline? | 0.02 | 0.04 | 0.06 | 0.07 | 0.02 | 1.00 |  |  |  |  |  |  |  |  |  |  |
| CON2: And AFTER speaking to the nurse, how confident were you in knowing how to manage the issue? | **.519^**^** | **.549^**^** | **.554^**^** | **.519^**^** | **.560^**^** | .202^**^ | 1.00 |  |  |  |  |  |  |  |  |  |
| CON3: How confident would you be in recommending the advice you were given to a friend who experiences similar? | **.435^**^** | **.449^**^** | **.453^**^** | **.447^**^** | **.511^**^** | 0.06 | .**649^**^** | 1.00 |  |  |  |  |  |  |  |  |
| EXT1: Was the information you were given what you EXPECTED to hear? | .185^**^ | .186^**^ | .160^**^ | .174^**^ | .276^**^ | .192^**^ | .206^**^ | .216^**^ | 1.00 |  |  |  |  |  |  |  |
| EXP1: Using the same scale, did the information provided by the helpline answer your query? | **.583^**^** | **.586^**^** | **.560^**^** | **.532^**^** | **.674^**^** | 0.04 | **.622^**^** | **.496^**^** | .280^**^ | 1.00 |  |  |  |  |  |  |
| EXP2: How USEFUL was the information or advice you received during your call? | **.586^**^** | **.589^**^** | **.587^**^** | **.556^**^** | **.643^**^** | 0.05 | **.636^**^** | **.550^**^** | **.317^**^** | **.745^**^** | 1.00 |  |  |  |  |  |
| ACT1: The nurse told me all the steps I needed to take. | **.331^**^** | **.354^**^** | **.398^**^** | **.373^**^** | **.394^**^** | -0.03 | **.465^**^** | **.340^**^** | 0.08 | **.465^**^** | **.472^**^** | 1.00 |  |  |  |  |
| ACT2: The nurse checked that I fully understood the information. | **.385^**^** | **.389^**^** | **.405^**^** | **.407^**^** | **.444^**^** | 0.04 | **.499^**^** | **.444^**^** | 0.08 | **.382^**^** | **.444^**^** | **.553^**^** | 1.00 |  |  |  |
| ACT3: After talking to the nurse I felt sure that I knew what to do. | **.407^**^** | **.405^**^** | **.463^**^** | **.435^**^** | **.415^**^** | 0.06 | **.628^**^** | **.420^**^** | 0.08 | **.489^**^** | **.525^**^** | **.537^**^** | **.666^**^** | 1.00 |  |  |
| SDM1: How much effort was made to listen to the things that mattered the most to you regarding the problem that you called about? | **.392^**^** | **.376^**^** | **.427^**^** | **.389^**^** | **.466^**^** | 0.02 | **.548^**^** | **.490^**^** | .143^**^ | **.467^**^** | **.512^**^** | **.413^**^** | **.374^**^** | **.429^**^** | 1.00 |  |
| SDM2: How much effort was made to help you understand the problem you called about? | **.488^**^** | **.493^**^** | **.512^**^** | **.464^**^** | **.583^**^** | 0.00 | **.572^**^** | **.535^**^** | .197^**^ | **.558^**^** | **.598^**^** | **.439^**^** | **.448^**^** | **.476^**^** | **.790^**^** | 1.00 |

**. Correlation is significant at the 0.01 level (2-tailed). *. Correlation is significant at the 0.05 level (2-tailed). Correlations ≥ .3 are in bold

HLQ: Health literacy questionnaire (having sufficient information to manage health); SATS: satisfaction; CON: confidence; EXT: expectations; EXP: caller experience; ACT: actionability; SDM: CollaboraRATE measure of shared decision making
